# Impact of Cannulation Strategy and Extracorporeal Blood Flow on Recirculation During Veno‐Venous Extracorporeal Membrane Oxygenation

**DOI:** 10.1111/aor.14961

**Published:** 2025-01-27

**Authors:** Jan‐Steffen Pooth, Jil Kristin Förster, Christoph Benk, Patric Diel, Sam Joé Brixius, Sven Maier, Alexander Supady, Tobias Wengenmayer, Dawid Leander Staudacher, Gerd‐Walter Haimerl, Martin Czerny, Julia Benk

**Affiliations:** ^1^ Department of Emergency Medicine Faculty of Medicine, University Medical Centre Freiburg, University of Freiburg Freiburg Germany; ^2^ Department of Cardiovascular Surgery Faculty of Medicine, University Medical Centre Freiburg, University of Freiburg Freiburg Germany; ^3^ Centre for Applied Simulation University of Applied Sciences Furtwangen Villingen‐Schwenningen Germany; ^4^ Interdisciplinary Medical Intensive Care Faculty of Medicine, University Medical Centre Freiburg, University of Freiburg Freiburg Germany

**Keywords:** acute respiratory distress syndrome, cannula, ECMO, extracorporeal circulation, recirculation, ultrasound dilution method, venovenous extracorporeal membrane oxygenation

## Abstract

**Introduction:**

Veno‐venous extracorporeal membrane oxygenation (V‐V ECMO) is increasingly used in the treatment of severe respiratory failure. Despite a significant increase in the worldwide use of extracorporeal lung assist devices recirculation remains a common complication and is associated with a reduced effectiveness of ECMO support and increased hemolysis. In this observational study we aimed to investigate the impact of cannula configuration and extracorporeal flow on recirculation.

**Materials and Methods:**

An observational retrospective study was performed, which included all patients, who received V‐V ECMO and recirculation measurements at the University Medical Center Freiburg between August 2021 and June 2023. Recirculation and extracorporeal flow were determined using ultrasonic indicator dilution technology. Patients were divided into subgroups according to their type of cannulation (dual lumen single‐site vs. bifemoral vs. femoro‐jugular).

**Results:**

A total of 215 recirculation measurements in 47 patients were performed. Dual lumen single‐site cannulation was associated with significantly lower recirculation rates (8.7% [0.0; 12.0]) compared to single lumen dual‐site cannulation (femoro‐jugular: 17.6% [0.0; 25.8]; bifemoral: 27.9% ± 13.4%). In addition, a positive linear correlation was observed between extracorporeal flow and recirculation in all subgroups. Recirculation increased significantly with rising extracorporeal flow in all subgroups.

**Conclusion:**

Recirculation is a common complication in V‐V ECMO and can lead to a reduction of ECMO effectiveness. Particular attention should be paid to optimal positioning of the cannulas in patients with more than one cannula. The ultrasonic indicator dilution method is a simple and quick method for measuring recirculation in V‐V ECMO and can be used at an early stage if effectiveness decreases.

## Graphical abstract

We conducted an observational study and compared recirculation measurements in V‐V ECMO. Recirculation and extracorporeal flow were determined using ultrasonic indicator dilution technology. Patients were divided into subgroups according to their type of cannulation (dual lumen single‐site vs. bifemoral vs. femoro‐jugular).

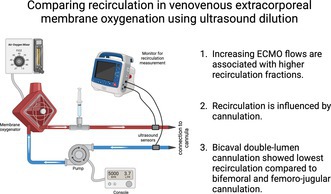

## 1 Introduction

The use of veno‐venous extracorporeal membrane oxygenation (V‐V ECMO) to acute respiratory failure in adults that is refractory to conventional therapies is increasing globally and increased particularly during the COVID‐19 pandemic [1]. The main objective of V‐V ECMO is to bridge hypoxic and hypercapnic phases in respiratory failure, thereby enabling recovery of pulmonary organ function and aiding recovery [2]. One common complication in V‐V ECMO is recirculation (e.g., due to cannula dislocation), which may reduce efficiency of ECMO support and thus ultimately result in hypoxemia, if no countermeasures are taken (e.g., cannula repositioning or increasing ECMO flow or increasing the fraction of inspired oxygen at the oxygenator) [2, 3, 4, 5, 6, 7, 8]. Therefore, it is crucial to minimize recirculation to ensure optimal oxygenation of the patient.

**FIGURE 1:**
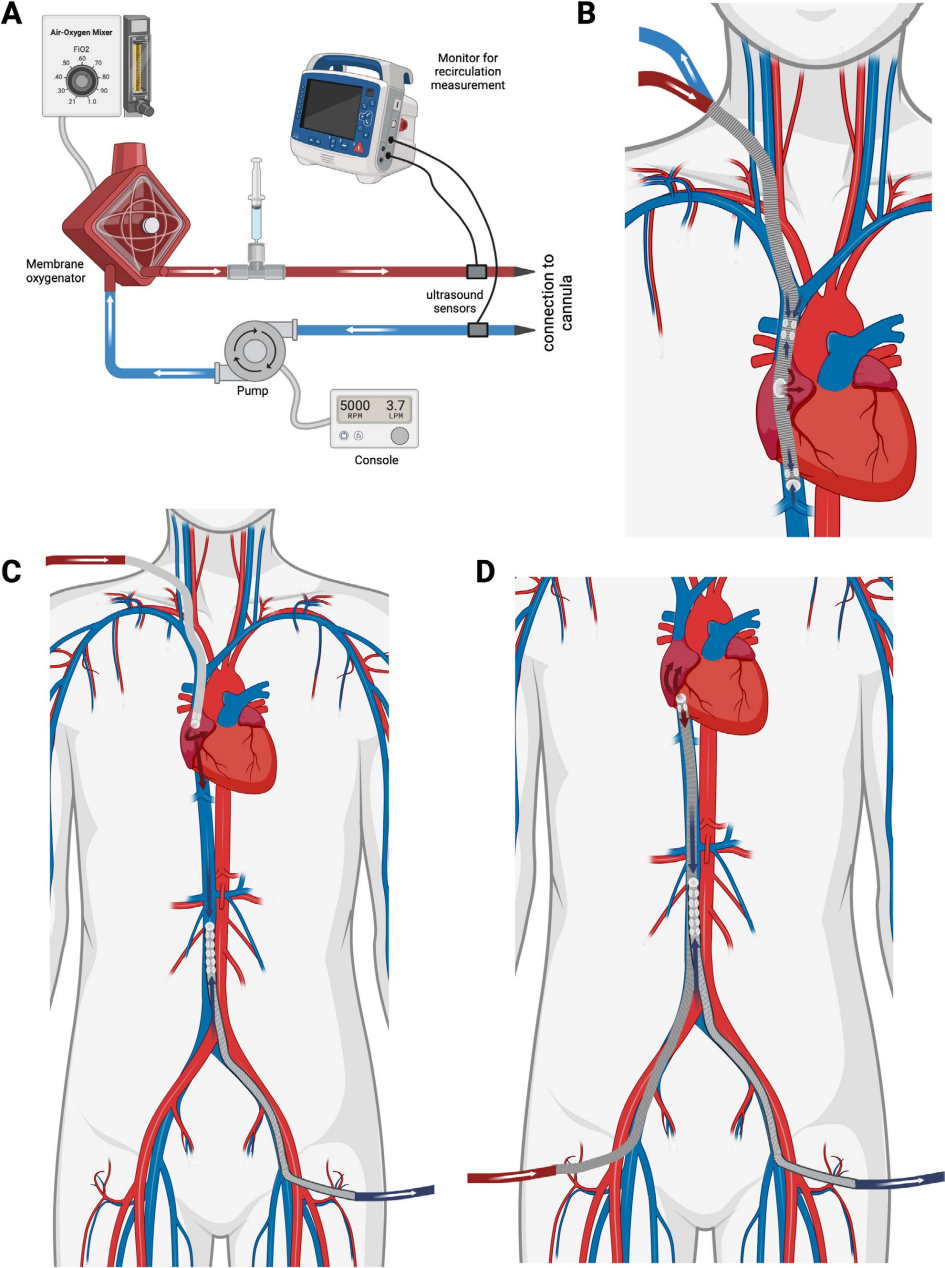
Measurement setup for determining recirculation (A). Schematics (B–D) show the cannula position and blood flow during dual lumen single‐site cannulation (B), femoro‐jugular cannulation (C) and bifemoral cannulation (D). [Color figure can be viewed at wileyonlinelibrary.com]

There are three main cannulation strategies that determine the choice of cannula for V‐V ECMO (Figure 1B–D): Dual lumen single‐site cannulation and single lumen cannula cannulation, which can be configurated in a bifemoral or a femoro‐jugular set‐up [2, 9]. Dual lumen single‐site cannulation involves inserting the cannula via the right jugular vein and advancing the tip through the right atrium into the inferior vena cava. The cannula is designed to drain blood from both the superior and inferior vena cava and to return it to the right atrium via an opening directed towards the tricuspid valve. Bifemoral venous cannulation involves positioning the tip of the drainage cannula in the vena cava below the diaphragm. The drained blood is then returned to the patient via the return cannula in the femoral vein on the opposite side, back into the right atrium. In case of femoro‐jugular cannulation with two single lumen cannulas, the blood is drained from the inferior vena cava through a cannula that is inserted via the femoral vein. It is recommended to position the cannula below the junction of the hepatic veins in adult patients [10]. The oxygenated blood is then returned to the patient via the internal jugular vein into the right atrium. All three strategies are commonly applied in clinical practice depending on the availability of material, anatomical conditions, the situation and the preference and experience of the treating team.

At the University Medical Center Freiburg, recirculation is measured using a differential transit time ultrasound and indicator dilution method [11]. This method quantifies the percentage of oxygen‐enriched blood reinfused via the ECMO circuit, which is withdrawn through the drainage cannula without passing the patient's bloodstream and has been repeatedly used in clinical practice to measure recirculation [8, 12, 13].

To improve the effectiveness of ECMO support through the improvement of oxygen delivery, we conducted a detailed analysis of the factors that had an influence on recirculation at our center.

## 2 Materials and Methods

The study was approved by the Ethics Committee of the Albert Ludwigs University of Freiburg (EK No. 23‐1256‐S1‐retro) and registered in the German Clinical Trials Register (DRKS00031890). The need to obtain an informed consent was waived by the ethics committee due to the observational and retrospective design of the study. Recirculation was measured using the HC101 ELSA monitor (Transonic Systems Inc., Ithaca, New York, USA). According to the manufacturer, the HC101 ELSA monitor has the following measuring accuracy: For measured values between 0% and 33%, the true value may deviate from the displayed value by ±5 percentage points. For measured values greater than 33%, the true value can deviate by up to 15% of the displayed value. An illustration of the measurement accuracy for different displayed values according to the manufacturer's specifications can be found in the online supplement (Figure S1). Figure 1 was created using Biorender [14].

### 2.1 Patient Population

This retrospective, monocentric, observational study examined ECMO recirculation in patients with V‐V ECMO at the University Medical Center Freiburg between August 2021 and June 2023. All patients ≥ 18 years who underwent recirculation measurement while on V‐V ECMO during the study period were included in this analysis. The patient cohort of 47 patients was divided into different study groups for statistical analysis based on cannulation strategy. Patients with dual lumen cannulation were cannulated with either Avalon Elite Bi‐Caval Dual Lumen Catheter (27–31 Fr., Getinge AB, Rastatt, Germany) or Crescent Jugular Dual Lumen Catheter (28–32 Fr., Medtronic GmbH, Meerbusch, Germany). Patients with single lumen cannulas received venous HLS cannulae (21–25 Fr., Getinge AB, Rastatt, Germany) and/or Bio‐Medicus venous cannulae (21–23 Fr., Medtronic GmbH, Meerbusch, Germany).

### 2.2 Recirculation Measurements

The HC101 ELSA monitor was used to measure recirculation, ECMO flow, and effective cardiac flow (ECF) through non‐invasive ultrasonic indicator dilution technology [12, 13]. Two ultrasound hemodilution sensors were connected in proximity to the drainage and the return cannula near the patient, ensuring proper alignment according to blood flow direction (Figure 1A). To conduct the measurement, a bolus of isotonic saline solution of 10 mL was administered into the post‐oxygenator port. This bolus acted as a marker, inducing a change in ultrasound velocity in the bloodstream, which was then used to determine recirculation [15].

### 2.3 Statistical Analysis

RStudio (version 2023.09.1 + 494, Boston, Massachusetts, USA) and R (version 4.3.2) were used for statistical analysis and data visualization [16]. Descriptive analyses were conducted to examine patient characteristics. To test for differences between the cannulation groups (Table 1), one‐way ANOVA was performed followed by Tukey's post hoc test. To analyze potential factors associated with recirculation, multiple regression analysis was performed (Table 2). Patient body mass index (BMI), ECMO flow, cannulation strategy (single lumen cannulas vs. dual lumen cannula), and days on ECMO were investigated as potential factors with an impact on recirculation in the multiple regression analysis. Furthermore, the correlation between ECMO flow and recirculation was visualized within each cannulation group and evaluated using Pearson's correlation coefficient. To control for the assumption of normally distributed data, Shapiro–Wilk test was performed. Normally distributed data were presented as mean ± standard deviation and otherwise as median and interquartile range (IQR). For related samples without normal distribution, the Wilcoxon test was used. *p*‐values less than 0.05 were considered statistically significant.

**TABLE 1:** Overview of the characteristics of the study population.

Cannulation strategy	Dual lumen single cannula	Single lumen cannulas		*p*
	Bicaval	Bifemoral	Femoro‐jugular	
Study population	27	9	11	NA
Measurements	109	44	62	NA
Survival [%]	70.4	55.5	54.5	0.54
Sex (m/f)	17/10	6/3	9/2	0.91
Age [years]	51.3 ± 11.2	60.8 ± 11.0	46.4 ± 10.1	0.12
Height [cm]	173.5 ± 11.5	171.3 ± 12.0	176.3 ± 11.2	0.85
Weight [kg]	99.5 ± 24.1	78.8 ± 18.4	94.7 ± 14.9	0.05
BMI [kg/m^2^]	33.1 ± 7.9	26.8 ± 5.2	30.7 ± 5.8	0.12
BSA [m^2^]	2.1 ± 0.3	1.9 ± 0.3	2.1 ± 0.2	0.08
Duration of ECMO [days]	29.5 [15.3; 45.8]	27.0 [9.5; 37.5]	36.0 ± 27.3	0.76
Duration on ICU [days]	45.0 [23.3; 57.5]	37.0 [18.0; 48.5]	52.6 ± 36.7	0.90

Abbreviations: BMI = body mass index; BSA = body surface area; f = female; ICU = intensive care unit; m = male; NA = not applicable.

**TABLE 2:** Results of the multiple regression analysis regarding factors with an influence on recirculation.

Factors	Estimate	95% CI	*p*
BMI	−0.0585	−0.2988, 0.1817	0.63
ECMO flow	0.0047	0.0032, 0.0061	< 0.001
Cannulation strategy: Use of single lumen cannulas	8.1277	3.9512, 12.3041	< 0.001
Days on ECMO	−0.1346	−0.2619, −0.0073	0.038

Abbreviations: BMI = body mass index; CI = confidence interval.

## 3 Results

We analyzed 215 recirculation measurements in 47 patients. Hundred and nine measurements were performed in 27 patients with dual lumen single‐site cannulation, 44 measurements in 9 patients with bifemoral cannulation, and 62 measurements in 11 patients with femoro‐jugular cannulation.

Within the entire study cohort, 46/47 patients (97.9%) were diagnosed with acute respiratory distress syndrome. The remaining patient required postoperative ECMO support following thoracic surgery with a lingulectomy.

The entire patient cohort had an average age of 51.6 ± 11.6 years, the mean BMI was 31.7 ± 7.3 kg/m^2^, and the average body surface area was 2.1 ± 0.3 m^2^. Patients spent a mean of 46.0 [20.5; 60.0] days in the intensive care unit with a mean duration of ECMO of 29.0 [13.0; 43.0] days.

No significant differences were observed among the investigated cannulation groups regarding baseline characteristics (Table 1). The repeated measurements of recirculation showed a constant measurement deviation of less than 10% over the entire measuring range.

Multiple regression analysis for the evaluation of factors affecting recirculation revealed that cannulation strategy (*p* < 0.001), ECMO flow (*p* < 0.001) and duration of ECMO (*p* = 0.038) had a significant impact on recirculation (Table 2).

### 3.1 Influence of Cannulation Strategy on Recirculation

**FIGURE 2:**
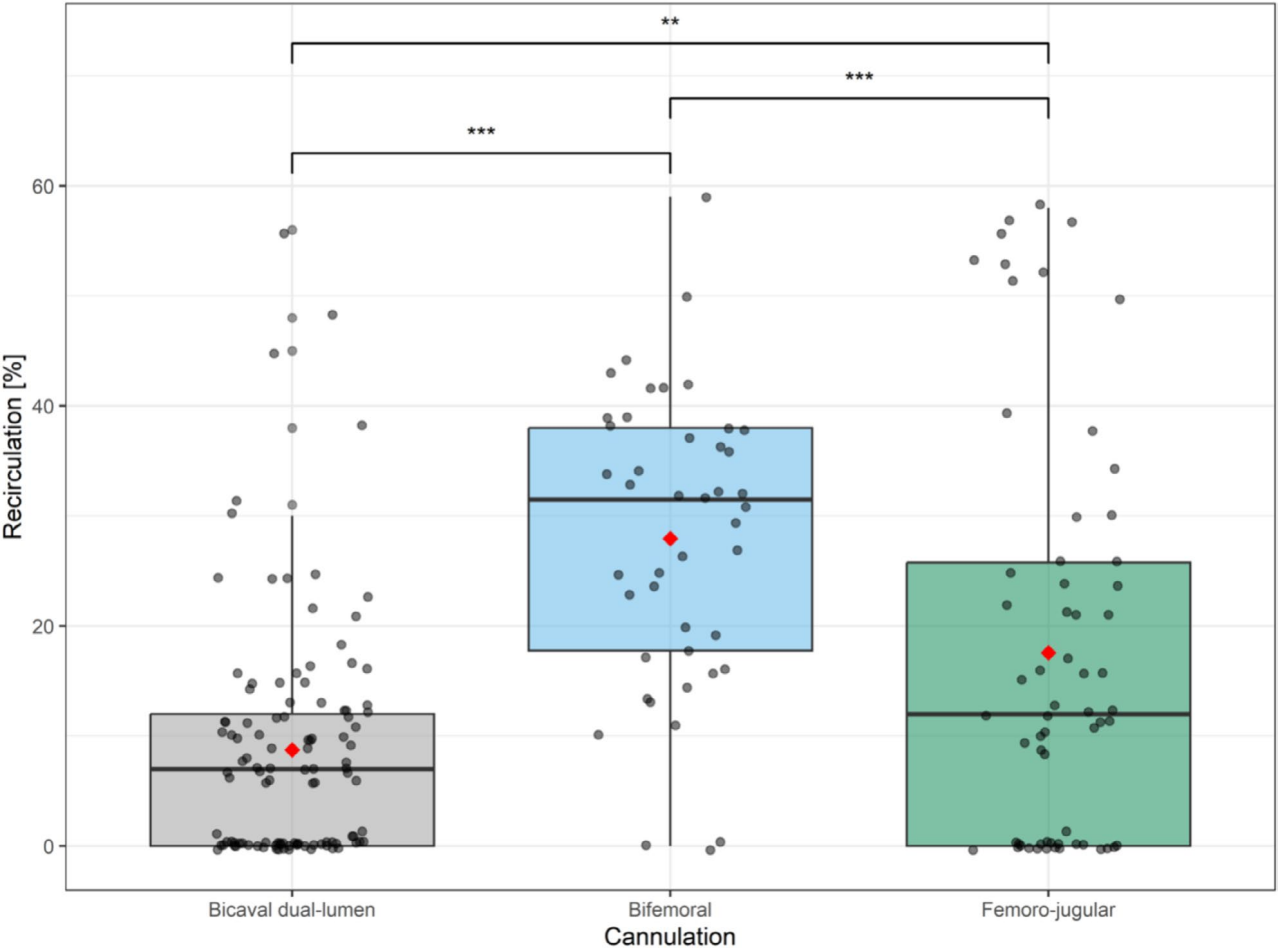
Average distribution of recirculation rates between cannulation groups. Red dot represents mean; **p < 0.01; ***p < 0.001. [Color figure can be viewed at wileyonlinelibrary.com]

Figure 2 shows recirculation rates in the different study groups.

Dual lumen single‐site (“bicaval”) cannulation exhibited the lowest recirculation rate, with a mean recirculation of 8.7% [0.0; 12.0]. In femoro‐jugular cannulation, a mean recirculation rate of 17.6% [0.0; 25.8] was detected and bifemoral cannulation was associated with the highest recirculation rate, averaging 27.9% (±13.4%). Thus, recirculation rates in bifemoral cannulation were on average 19.2% higher (95%‐confidence interval, CI [13.3; 25.5]) and in femoro‐jugular cannulation 10.4% (95% CI [3.9; 16.9]) higher than recirculation rates in dual lumen single‐site cannulation. Additionally, femoro‐jugular cannulation exhibited on average an 8.8% higher recirculation compared to dual lumen cannulation (95% CI [3.6; 14.1]).

**FIGURE 3:**
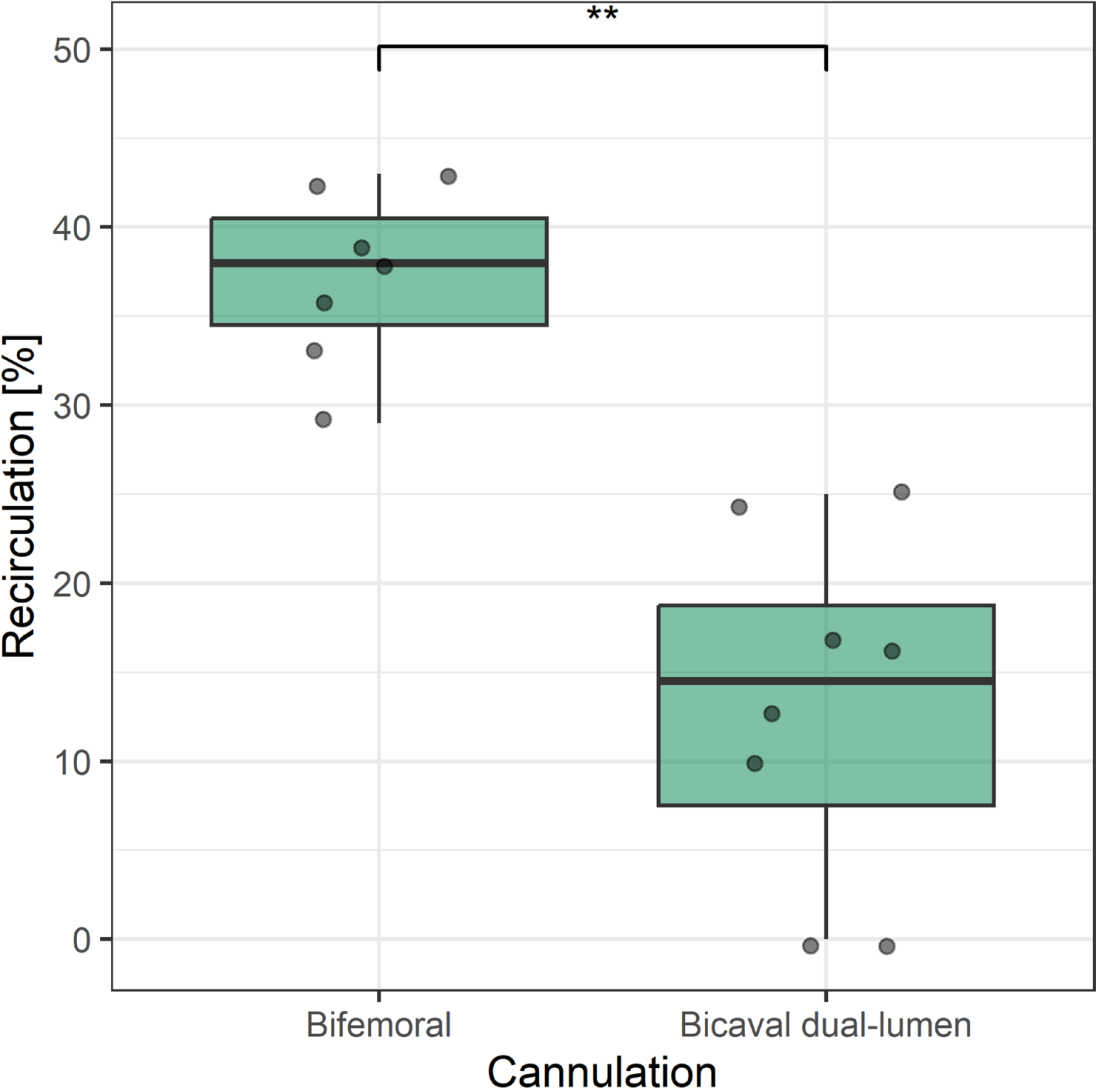
Comparison of bifemoral cannulation with bicaval dual lumen cannulation in one patient; **p < 0.01. [Color figure can be viewed at wileyonlinelibrary.com]

The effect of the cannulation strategy on recirculation was even more evident in one patient, who underwent a conversion from initial bifemoral cannulation to dual lumen cannulation. Figure 3 shows the recirculation measurements before and after conversion. Initially under bifemoral cannulation an average recirculation of 37.1% was measured with a measured ECF between 3.8 and 4.5 L/min at an ECMO flow rate of 6.2–6.8 L/min. After conversion to dual lumen cannulation, recirculation in this patient was 13.1% accompanied by an ECF ranging from 3.0 to 4.3 L/min and an ECMO flow rate of 3.8–4.3 L/min. In this case, the median recirculation was 14.5% [7.5; 18.8] for dual lumen cannulation and 38% [34.5; 40.5] for bifemoral cannulation.

### 3.2 Effect of ECMO Flow on Recirculation

**FIGURE 4:**
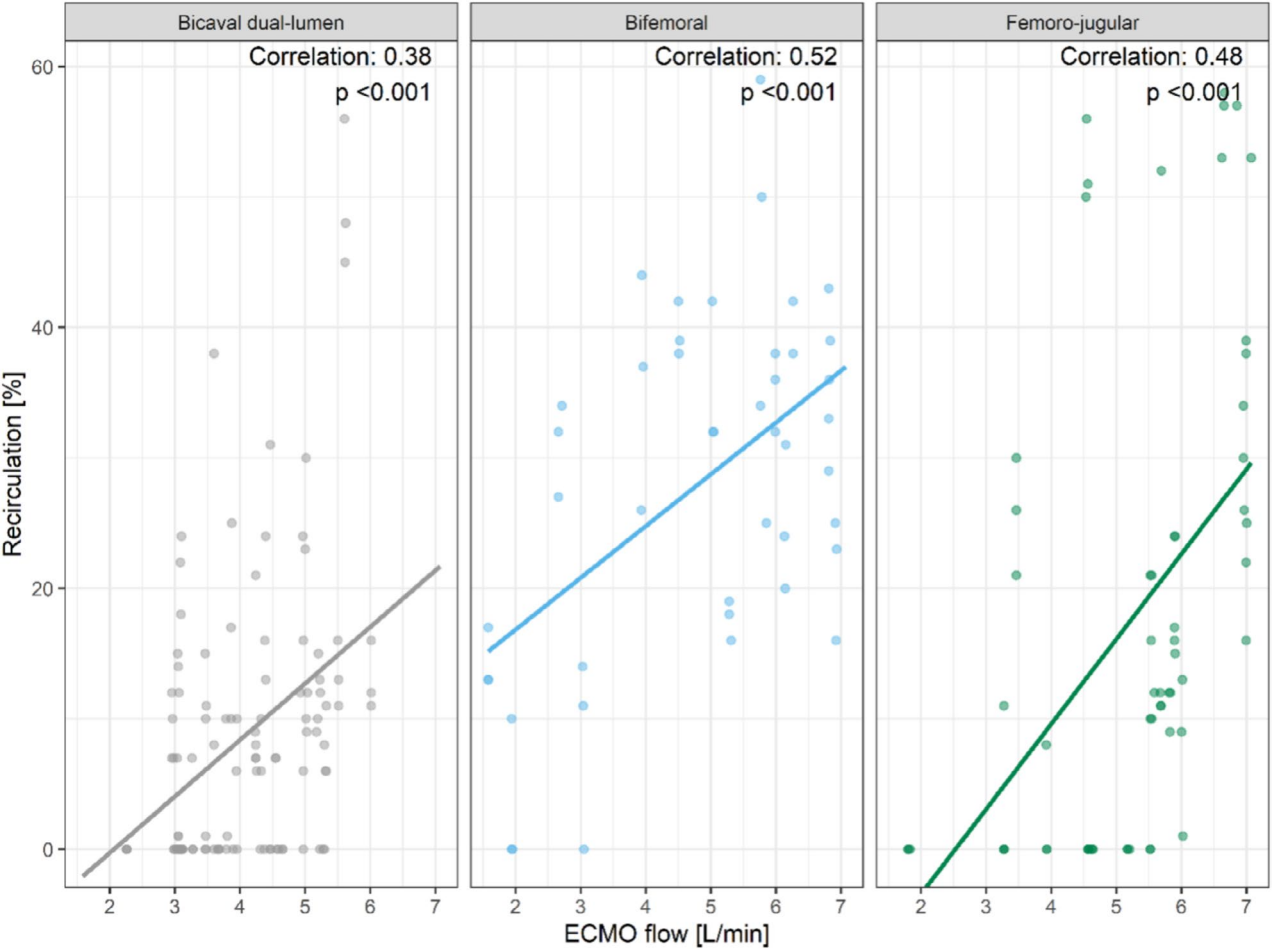
Impact of ECMO flow on recirculation within the different cannulation groups. [Color figure can be viewed at wileyonlinelibrary.com]

Since ECMO flow was also identified to have a significant effect on recirculation, recirculation measurements were plotted against the individual ECMO flow rates in each cannulation group (Figure 4). A significant positive linear correlation was observed between recirculation and ECMO flow in all cannulation groups (*p* < 0.001).

Higher ECMO flows were associated with increased recirculation. The correlation was more evident in patients with bifemoral cannulation, where a correlation coefficient of 0.52 was found. The lowest correlation was observed in patients with dual lumen cannulation, with a correlation coefficient of 0.38, while in patients with femoro‐jugular cannulation a correlation coefficient of 0.48 was detected (Figure 4).

### 3.3 Effect of Duration of ECMO on Recirculation

**FIGURE 5:**
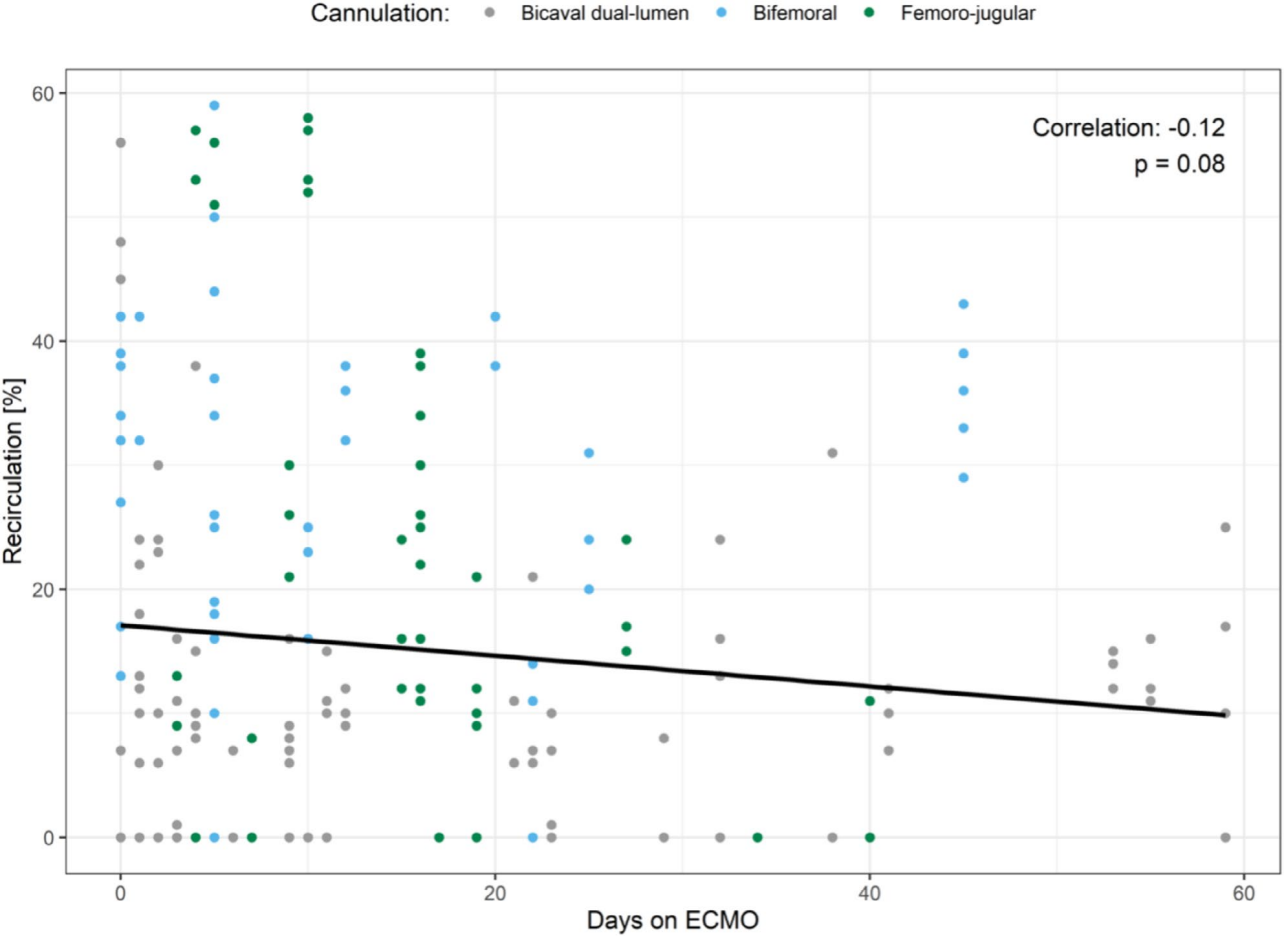
Effect of duration of ECMO in days on recirculation. [Color figure can be viewed at wileyonlinelibrary.com]

According to multiple regression analysis, days on ECMO had a significant influence on recirculation. For illustration, we plotted recirculation measurements against days on ECMO (Figure 5). This showed a weak negative correlation of −0.12 without statistical significance (*p* = 0.08). A closer analysis revealed a significant interaction between days on ECMO and ECMO flow in relation to recirculation (*p* = 0.02).

## 4 Discussion

Recirculation (i.e., oxygenated blood the patient received from the ECMO circuit is directly drained back to the ECMO circuit) significantly reduces the effectiveness of V‐V ECMO and may contribute to severe hypoxemia, hemolysis and end organ damage [2, 6, 10, 17]. Notably, Gehron and colleagues showed that an increase in ECMO flow is associated not only with an increase in recirculation, but also with an increase in oxygen delivery [8]. It should therefore be noted that increased recirculation is not automatically associated with the risk of hypoxemia. However, if the efficiency of an ECMO system is reduced while the flow remains constant, recirculation must be considered and its possible causes (e.g., cannula dislocation) should be investigated. It is therefore crucial to recognize increasing recirculation at an early stage of organ support in order to take possible countermeasures as soon as possible.

In this study, we identified cannulation strategy and ECMO flow as major contributors to recirculation. Within each cannulation group, we observed a linear relationship between recirculation and extracorporeal blood flow (see Figure 4). Our results confirm previous findings that less recirculation can be detected when using a bicaval dual lumen cannula in V‐V ECMO compared to bifemoral or femoro‐jugular cannulation [4, 18, 19]. In 2016, Jaywardene and colleagues investigated recirculation with different cannulation configurations in a mock circulation loop for extracorporeal circulation. In their mock loop the least recirculation was also observed using a dual lumen cannula [20].

This is probably partly due to the design of dual lumen cannulas. In a dual lumen cannula, there is already an optimized set‐up with a fixed distance between the inflow and outflow opening. This underlines the particular importance of cannula positioning in V‐V ECMO.

### 4.1 Cannulation Configuration and Positioning

Particularly in V‐V ECMO with two single lumen cannulas, drainage, and extracorporeal flow are highly affected by positioning. Javidfar and colleagues were able to show that pointing the tip of the return cannula towards the tricuspid valve and away from the drainage cannula opening can reduce recirculation [21]. This position facilitates direct blood flow into the right ventricle during diastole by avoiding proximity to the drainage cannula opening. According to their work, deep insertion of the return cannula is not necessary when properly orientated towards the tricuspid valve [22]. However, colleagues from Sweden were able to report data on double lumen cannulas and advise against rotation of inserted double lumen cannulas if the insertion depth is sufficient [18]. In general, their findings are consistent with our recirculation measurements using a bicaval dual lumen cannula and underline the importance to differentiate in between cannulation configurations. Especially in case of bifemoral cannulation, the openings of the drainage and the return cannula are placed in the same vessel facing in the same direction, facilitating the drainage of reinfused blood. A reduction in recirculation can be achieved by adjusting the cannula position, such as retracting one or both cannulas to increase the distance between the drainage and the return cannula [4].

Another contributing factor for the high recirculation rate in bifemoral cannulation is definitely the presence of numerous proximal openings on the femoral return cannula, which reduce the distance between drainage and return cannula and can facilitate recirculation. Rauh and colleagues were able to show that blood flow is not equally distributed between these side‐holes of the cannula, making an optimal positioning of the drainage cannula in bifemoral cannulation even more difficult [23].

### 4.2 ECMO Flow

As shown by the calculation of the Reynolds number, the flow behavior of a fluid depends on the inner diameter of the tubes, the flow velocity, and the kinematic viscosity [24]. Accordingly, at higher flow rates, more vortices occur, resulting in a turbulent flow. Researchers from Louisiana State University and the University of Kentucky developed a computational fluid dynamics model to simulate blood flow during filling and emptying of the right atrium at various circulatory flows in V‐V ECMO [22]. Their study showed that at low ECMO flows, reinfused blood mixes in the upper part of the atrium during right atrial filling and does not reach the drainage cannula due to cannula positioning. They also observed that in simulations with higher ECMO flows, more reinfused blood reached the drainage cannula due to blood turbulence, and recirculation rates of up to 40% were achieved when cardiac output was matched by extracorporeal flow. Their simulations align with our observations in this study (see Figure 4).

### 4.3 Clinical Relevance

It should be noted that survival did not differ in between groups in this study (see Table 1). Since the reported data set only included patients with recirculation measurements, we would like to point out that larger studies from our center were also unable to report a survival benefit associated with dual‐lumen cannulation [25]. Nevertheless, if the efficiency of an ECMO system decreases while the flow remains constant, recirculation must be considered and its possible causes (e.g., cannula dislocation) should be investigated. The ultrasound dilution method can be used for early detection of increased recirculation, but its results need to be interpreted within the clinical context. In case of uncertainty and/or high recirculation values, it is advisable to validate the recirculation measurement, for example by means of central venous saturation measurement [26].

### 4.4 Limitations

The present study is a monocentric, retrospective observational study. The study design was not randomized and therefore did not account for possible differences in baseline characteristics of the patient population. Another limitation of the study is the small number of bifemoral and femoro‐jugular cannulated patients, which may increase the variability of the results and limit the detection of further factors, which may have an influence on recirculation. Furthermore, the number of different cannula sizes in each group was too small for any meaningful evaluation. We acknowledge that cannula size may influence recirculation. Since this was a retrospective study, it was impossible to include cardiac output data in the analysis, as it was not measured routinely in these patients. It has already been shown that a higher cardiac output is associated with lower recirculation [8]. Therefore, we believe that an improvement in cardiac function during the ICU stay may have been a reason for the observed decrease in recirculation with increasing duration on ECMO. We were unfortunately unable to correct for this confounder.

## 5 Conclusion

In this study, the different effects of cannulation strategy and ECMO flow on recirculation in V‐V ECMO were investigated. We were able to detect a highly significant effect of extracorporeal flow and cannulation configuration on recirculation. Increasing ECMO flows were associated with higher recirculation fractions. Recirculation was also influenced by the choice of cannulation strategy, with the bicaval double‐lumen cannulation strategy showing a significant reduction compared to bifemoral and femoro‐jugular cannulation methods. The ultrasonic indicator dilution method is a simple and quick method for measuring recirculation in V‐V ECMO and can be used at an early stage if effectiveness decreases. Adjusting cannula position can decrease recirculation and enhance efficacy of ECMO support.

## Author Contributions

J.‐S.P., J.K.F., S.M., and J.B. planned and designed the study. J.K.F., P.D., S.M. were involved in data collection. J.‐S.P., J.K.F., P.D., and G.‐W.H. analyzed the data. J.K.F. visualized the data and performed all statistical analyses. J.‐S.P., J.K.F., S.J.B., and J.B. drafted the original manuscript. C.B., S.M., and G.‐W.H. were involved in the interpretation of data. A.S., D.L.S., and S.M. were major contributors in the revision of the manuscript. C.B., T.W., and C.M. provided clinical resources and funding. All authors read and approved the final manuscript.

## Conflicts of Interest

M.C. is a consultant to Terumo Aortic, Medtronic, Endospan, and NEOS, received speaking honoraria from Cryolife‐Jotec, and is a shareholder of TEVAR Ltd. and Ascense Medical. J.S.P., S.J.B., and C.B. are part‐time employees of Resuscitec GmbH, Freiburg, Germany, which is a start‐up company from the University Medical Centre Freiburg. A.S. received speaker's honoraria and research grants from CytoSorbents, speaker's honoraria from Getinge and AstraZeneca, and consulting honoraria and travel support from ArtCline.

## Supporting information


**Figure S1.** Visualization of the accuracy of recirculation measurements by HC101 ELSA monitor as reported by its manufacturer.

## Data Availability

All analyzed datasets are available in anonymous form from the corresponding author upon reasonable request.

## References

[1] J. E. Tonna , P. S. Boonstra , G. MacLaren , et al., “Extracorporeal Life Support Organization Registry International Report 2022: 100,000 Survivors,” ASAIO Journal 70 (2024): 131–143. 38181413 10.1097/MAT.0000000000002128 PMC10962646

[2] J. E. Tonna , D. Abrams , D. Brodie , et al., “Management of Adult Patients Supported With Venovenous Extracorporeal Membrane Oxygenation (VV ECMO): Guideline From the Extracorporeal Life Support Organization (ELSO),” ASAIO Journal 67 (2021): 601–610. 33965970 10.1097/MAT.0000000000001432 PMC8315725

[3] A. Xie , T. D. Yan , and P. Forrest , “Recirculation in Venovenous Extracorporeal Membrane Oxygenation,” Journal of Critical Care 36 (2016): 107–110. 27546757 10.1016/j.jcrc.2016.05.027

[4] D. Abrams , M. Bacchetta , and D. Brodie , “Recirculation in Venovenous Extracorporeal Membrane Oxygenation,” ASAIO Journal 2015, no. 61 (1992): 115–121. 10.1097/MAT.0000000000000179 25423117

[5] O. Palmér , K. Palmér , J. Hultman , and M. Broman , “Cannula Design and Recirculation During Venovenous Extracorporeal Membrane Oxygenation,” ASAIO Journal 2016, no. 62 (1992): 737–742. 10.1097/MAT.0000000000000440 PMC5098462 27660904

[6] L. P. Parker , A. S. Marcial , T. B. Brismar , L. M. Broman , and L. Prahl Wittberg , “Cannulation Configuration and Recirculation in Venovenous Extracorporeal Membrane Oxygenation,” Scientific Reports 12 (2022): 16379. 36180496 10.1038/s41598-022-20690-x PMC9523655

[7] A. Zanella , D. Salerno , V. Scaravilli , et al., “A Mathematical Model of Oxygenation During Venovenous Extracorporeal Membrane Oxygenation Support,” Journal of Critical Care 36 (2016): 178–186. 27546769 10.1016/j.jcrc.2016.07.008

[8] J. Gehron , D. Bandorski , K. Mayer , and A. Böning , “The Impact of Recirculation on Extracorporeal Gas Exchange and Patient Oxygenation During Veno‐Venous Extracorporeal Membrane Oxygenation—Results of an Observational Clinical Trial,” Journal of Clinical Medicine 12 (2023): 416. 36675344 10.3390/jcm12020416 PMC9866780

[9] J. A. Lindholm , “Cannulation for Veno‐Venous Extracorporeal Membrane Oxygenation,” Journal of Thoracic Disease 10 (2018): S606–S612. 29732177 10.21037/jtd.2018.03.101 PMC5911563

[10] L. Beck , M. Burg , W. Heindel , and C. Schülke , “Extrakorporale Membranoxygenierung bei Erwachsenen—Varianten, Komplikationen unter Therapie und die Rolle der radiologischen Diagnostik,” RoFo Fortschritte auf dem Gebiete der Rontgenstrahlen und der Nuklearmedizin 189 (2016): 119–127. 28033607 10.1055/s-0042-118885

[11] C. Sreenan , H. Osiovich , P.‐Y. Cheung , and R. P. Lemke , “Quantification of Recirculation by Thermodilution During Venovenous Extracorporeal Membrane Oxygenation,” Journal of Pediatric Surgery 35 (2000): 1411–1414. 11051139 10.1053/jpsu.2000.16402

[12] A. F. van Heijst , F. H. van der Staak , A. F. de Haan , et al., “Recirculation in Double Lumen Catheter Veno‐Venous Extracorporeal Membrane Oxygenation Measured by an Ultrasound Dilution Technique,” ASAIO Journal 2001, no. 47 (1992): 372–376. 10.1097/00002480-200107000-00015 11482489

[13] D. Clements , J. Primmer , P. Ryman , B. Marr , B. Searles , and E. Darling , “Measurements of Recirculation During Neonatal Veno‐Venous Extracorporeal Membrane Oxygenation: Clinical Application of the Ultrasound Dilution Technique,” Journal of Extra‐Corporeal Technology 40 (2008): 184–187. 18853830 PMC4680644

[14] J.‐S. Pooth , Created in BioRender, BioRender.com/h06f428 2023.

[15] T. A. Depner , N. M. Krivitski , and D. MacGibbon , “Hemodialysis Access Recirculation Measured by Ultrasound Dilution,” ASAIO Journal 41 (1995): M749–M753. 8573907 10.1097/00002480-199507000-00113

[16] R Core Team , R: A Language and Environment for Statistical Computing, https://www.R‐project.org/. (2020).

[17] R. P. Barbaro , G. MacLaren , P. S. Boonstra , et al., “Extracorporeal Membrane Oxygenation Support in COVID‐19: An International Cohort Study of the Extracorporeal Life Support Organization Registry,” Lancet 396 (2020): 1071–1078. 32987008 10.1016/S0140-6736(20)32008-0 PMC7518880

[18] L. P. Parker , A. Svensson Marcial , T. B. Brismar , L. M. Broman , and L. Prahl Wittberg , “Hemodynamic and Recirculation Performance of Dual Lumen Cannulas for Venovenous Extracorporeal Membrane Oxygenation,” Scientific Reports 13 (2023): 7472. 37156961 10.1038/s41598-023-34655-1 PMC10167322

[19] D. Wang , X. Zhou , X. Liu , B. Sidor , J. Lynch , and J. B. Zwischenberger , “Wang‐Zwische Double Lumen Cannula‐Toward a Percutaneous and Ambulatory Paracorporeal Artificial Lung,” ASAIO Journal 2008, no. 54 (1992): 606–611. 10.1097/MAT.0b013e31818c69ab 19033774

[20] I. D. Jayewardene , A. Xie , A. Iyer , R. Pye , and K. Dhital , “Development of a Mock Extracorporeal Membrane Oxygenation Circuit to Assess Recirculation,” ASAIO Journal 2016, no. 62 (1992): 496–497. 10.1097/MAT.0000000000000350 26809084

[21] J. Javidfar , D. Wang , J. B. Zwischenberger , et al., “Insertion of Bicaval Dual Lumen Extracorporeal Membrane Oxygenation Catheter With Image Guidance,” ASAIO Journal 2011, no. 57 (1992): 203–205. 10.1097/MAT.0b013e3182155fee 21499077

[22] S. A. Conrad and D. Wang , “Evaluation of Recirculation During Venovenous Extracorporeal Membrane Oxygenation Using Computational Fluid Dynamics Incorporating Fluid‐Structure Interaction,” ASAIO Journal 2021, no. 67 (1992): 943–953. 10.1097/MAT.0000000000001314 PMC8318564 33315664

[23] P. Rauh , C. Benk , F. Beyersdorf , and M. Russe , “Determination of Local Flow Ratios and Velocities in a Femoral Venous Cannula With Computational Fluid Dynamics and 4D Flow‐Sensitive Magnetic Resonance Imaging: A Method Validation,” Artificial Organs 45 (2021): 506–515. 33185904 10.1111/aor.13859

[24] O. Reynolds , “XXIX. An Experimental Investigation of the Circumstances Which Determine Whether the Motion of Water Shall Be Direct or Sinuous, and of the Law of Resistance in Parallel Channels,” Philosophical Transactions of the Royal Society of London 174 (1883): 935–982.

[25] C. Noe , F. A. Rottmann , X. Bemtgen , A. Supady , T. Wengenmayer , and D. L. Staudacher , “Dual Lumen Cannulation and Mobilization of Patients With Venovenous Extracorporeal Membrane Oxygenation,” Artificial Organs 47 (2023): 1654–1662. 37358935 10.1111/aor.14604

[26] E. M. Darling , T. Crowell , and B. E. Searles , “Use of Dilutional Ultrasound Monitoring to Detect Changes in Recirculation During Venovenous Extracorporeal Membrane Oxygenation in Swine,” ASAIO Journal 2006, no. 52 (1992): 522–524. 10.1097/01.mat.0000237589.20935.a4 16966850

