# Supplementary figures and images for: Impact of Cannulation Strategy and Extracorporeal Blood Flow on Recirculation During Veno‐Venous Extracorporeal Membrane Oxygenation
Source: Artif Organs. 2025 Jan 27;49(6):1012–20. doi: 10.1111/aor.14961 (PMC12120813; doi:10.1111/aor.14961)

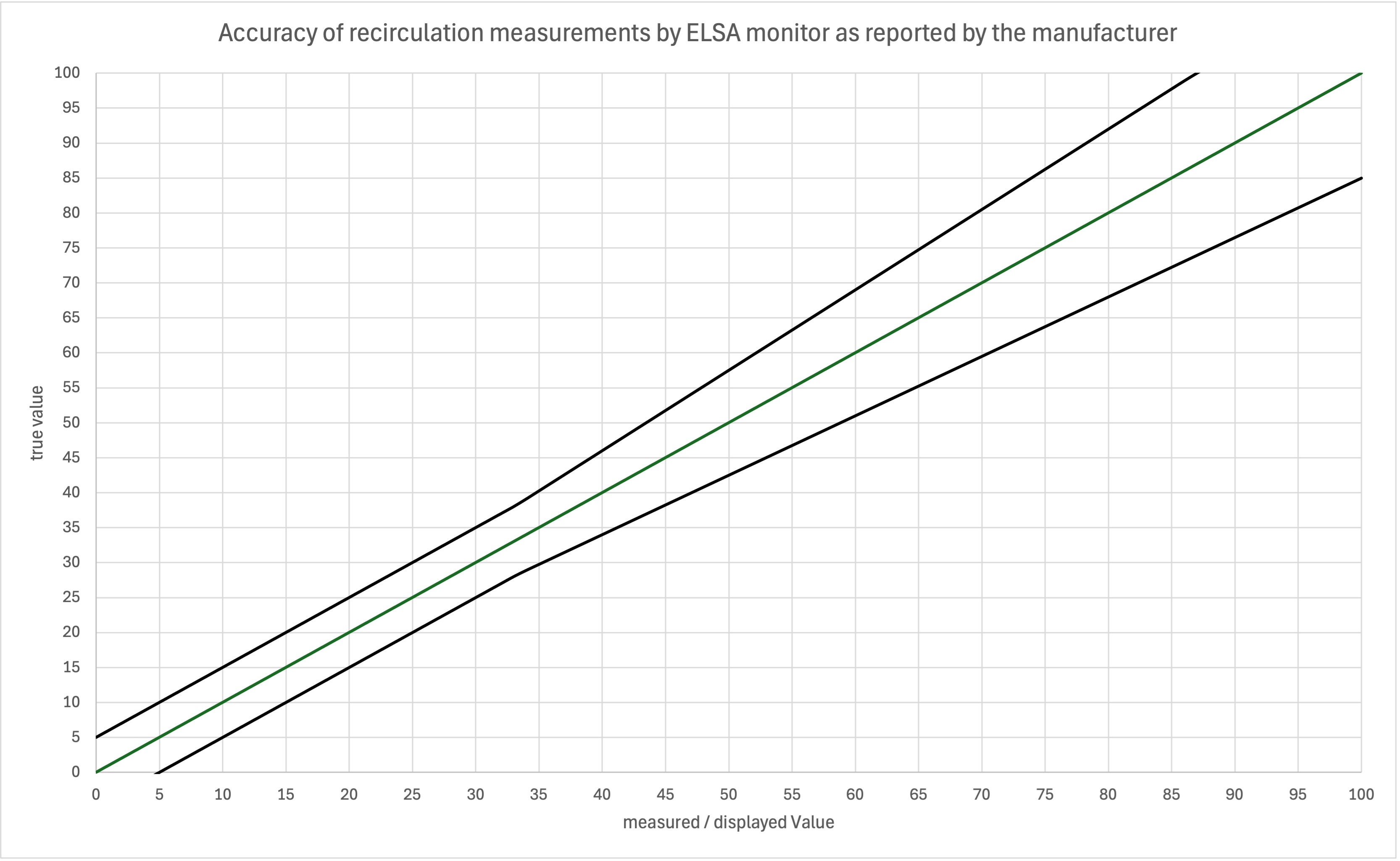

Supplement: Supplementary file 1 — Figure S1. Visualization of the accuracy of recirculation measurements by HC101 ELSA monitor as reported by its manufacturer. [file AOR-49-1012-s001.png]
